# CEDART Study: protocol for a non-randomized feasibility study

**DOI:** 10.21203/rs.3.rs-6378596/v1

**Published:** 2025-05-13

**Authors:** Jung Kwak, Aveen Jafari, Allison Salter, Andrea Perry, Alexis de Montfort Shepherd, Menaka Jayasundera, Sarah Stayer, Mike Brode, Snehal Patel, Elizabeth Kvale

**Affiliations:** The University of Texas at Austin School of Nursing; The University of Texas at Austin; The University of Texas at Austin; Raymond G Murphy VA Medical Center: New Mexico VA Health Care System; The University of Texas at Austin Dell Medical School; The University of Texas at Austin Dell Medical School; The University of Texas at Austin Dell Medical School; The University of Texas at Austin Dell Medical School; The University of Texas at Austin Dell Medical School; Baylor College of Medicine

**Keywords:** persons with dementia, caregiver, care transition, community health worker, psychosocial intervention, non-randomized feasibility study

## Abstract

**Background:**

Family caregivers of persons with dementia face significant challenges in navigating hospital-to-community transitions. Community health worker-led interventions offer a promising approach to delivering culturally responsive psychosocial support and care navigation. However, there is limited evidence on the feasibility of implementing care transition interventions for this population across healthcare settings. This single-site, single-arm pilot study evaluates the feasibility, acceptability, and preliminary impact of a community health worker-led care transition intervention for caregivers of persons with dementia, Connecting and Empowering Persons living with Dementia and cARegivers during Transition (CEDART).

**Methods:**

Forty hospitalized persons with dementia and their caregivers will receive the CEDART intervention. Eligible patients with confirmed or suspected dementia will be enrolled from a Texas hospital. Respective caregivers will then participate in an initial assessment meeting followed by individualized care planning and follow-up meetings led by the community health worker. The community health worker will continue to provide caregivers with tailored dementia care education, emotional support, and connections to community resources for up to three months post-discharge. Input from a community advisory board of hospital system partners, community-based service providers, and dementia care experts - alongside key informant interviews with hospital providers, community providers, and caregiver participants receiving CEDART intervention, will be used to evaluate the acceptability of the intervention. Key informant interviews will be conducted to assess whether the intervention meets and addresses stakeholder priorities. Participating caregivers will complete baseline, 6-week, and 12-week follow-up interviews to assess caregiver outcomes and intervention feasibility and acceptability.

**Conclusions:**

Study findings will be used to evaluate the feasibility and acceptability of the proposed CEDART care transition intervention for persons with dementia and family caregivers. These results will inform the design of a future definitive randomized control trial.

**Trial registration:**

NCT06831318 (registered and posted on ClinicalTrials.gov)

## Background

Transitions of care refer to the process of moving patients between health care practitioners, various care settings, and home as their care needs change [1]. Hospital-based care transitions (i.e., leaving the hospital for care elsewhere) pose significant risks for patients’ safety [2]. Most notably, persons with dementia are 1.5 – 2 times more likely than non-dementia peers to be hospitalized [3, 4]. This heightened risk can make the care transition process especially fraught and challenging. For persons with dementia, such transitions often involve short-term stays in skilled nursing facilities before returning home [5]. With each transfer from one care setting to another, the risk of medical errors, incongruent or unnecessary medical treatments, and hospital readmission increases [6–8]. During these transitions, family caregivers play crucial roles in the care of persons with dementia [9, 10]. Thus, it is critical to engage and collaborate with family caregivers to ensure safe care transitions across healthcare settings.

Transitional care interventions include the delivery of time-limited services to promote continuity and coordination of healthcare. These interventions have been shown to improve patient outcomes across diverse health conditions, including heart failure, chronic obstructive pulmonary disease, acute myocardial infarction, and stroke [11–17]. However, persons with dementia have been underrepresented in this body of research. Although many assume that the question of how to improve care transition outcomes has been answered, transitional care interventions are underutilized or insufficiently adapted for persons with dementia and their family caregivers [9, 18]. Current transitional care models for persons with dementia are limited in number and tend to be broadly designed, often involving complex, multiple TC support types (i.e., patient education, medication reconciliation, referral to community-resources, home visits) delivered over extended periods (e.g., months to years). Some hospital-based transitional care models have shown efficacy for persons with dementia[19], but their complexity and resource demands limit scalability and wider adoption.

To address these challenges, we developed our hospital-based care transition intervention, **C**onnecting and **E**mpowering Persons living with **D**ementia and c**AR**egivers during **T**ransition (CEDART), to be delivered by a community health worker for use in a future definitive randomized control trial. Community health workers [20–22] are lay community members who work—either paid or voluntarily—with local health systems in urban and rural settings. Community health workers are viewed as trusted allies of their respective communities through shared ethnicity, language, socioeconomic background, and/or experiences with those they serve. Thus, they can effectively bridge cultural gaps between vulnerable patients, family caregivers, and the healthcare system to holistically address caregiving needs [23–28]. Community health workers also have the capacity to provide pragmatic caregiving advice, information, and individualized support for family caregivers, thereby fostering their self-efficacy and resilience. Given their role as resource connectors and trusted community members, community health workers are equipped to deliver culturally responsive, effective care transition interventions. Our community health worker-led approach is further informed by the Sociocultural Stress and Coping Model [29], Roberts’ Crisis Intervention Model [30], and evidence from the literature on effective components of existing care transition models [16].

## Methods

### Study aim

This study will evaluate feasibility and inform the optimal design of a future definitive randomized trial to test the efficacy of the CEDART intervention for persons with dementia and their caregivers during care transitions.

### Objectives

1. To assess the feasibility and acceptability of the proposed CEDART intervention to participants.
2. To test the feasibility and acceptability of a future definitive trial.

The main hypotheses of the study are:

1. The community health worker interventionist will adhere to the intervention protocol with a score of 70% or higher on the intervention fidelity checklist throughout the delivery period.
2. Caregiver ratings of our intervention will show that the community health worker interventionist was helpful and satisfactory at the end of the intervention period.
3. The intervention will be feasible, as evidenced by intervention completion rates greater than or equal to 70%. Completion will be defined by the proportion of participants who complete follow-up sessions and complete assessments at baseline, 6-week, and 12-week time points.
4. Patient and caregiver outcomes will show improvement over time, as indicated by positive trends in follow-up data at 6 and 12 weeks compared to baseline.

### Design

**Figure 1:**
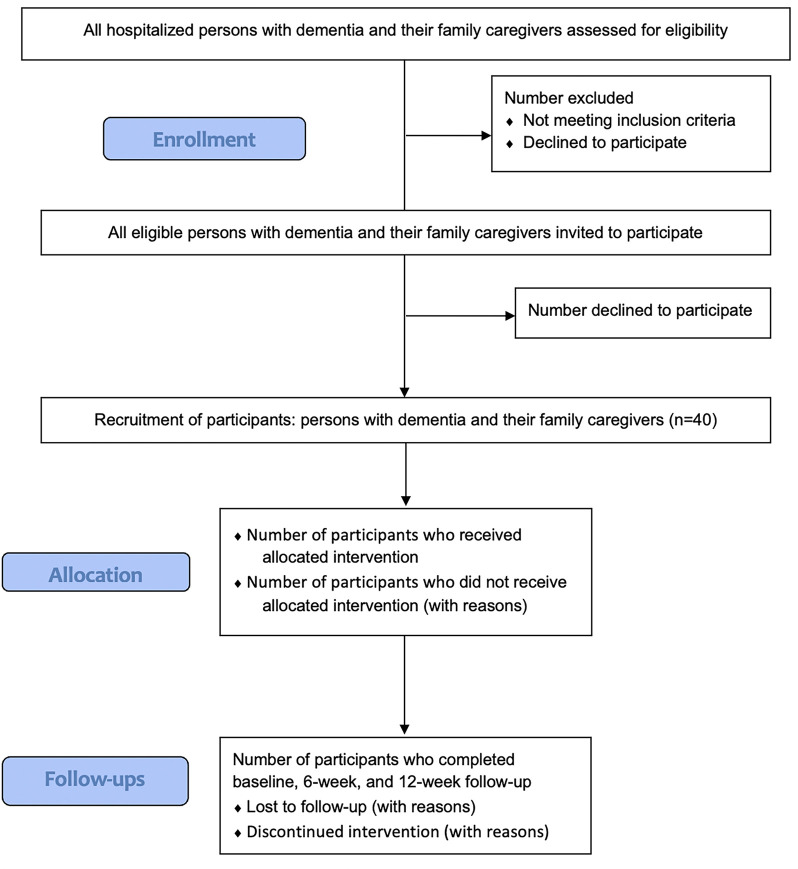
Participant Flow Diagram per CONSORT 2010

This study will use a nonrandomized feasibility design and will follow the SPIRIT 2013 [31] guidelines and reporting template [see Additional file 1]. Enrollment, intervention, and assessment timelines are displayed in Table 1. The CONSORT extension guidelines and checklists were also adapted for the purpose of the study protocol [see Additional File 2]. The CONSORT flow diagram in Figure 1 depicts the participant flow in our intervention.

Specifically, the study is designed as a single-site, single-arm feasibility study with 40 hospitalized patient-caregiver dyads receiving the CEDART intervention over a three-month period. All hospitalized dyads will be recruited from a medical school-affiliated hospital located in Austin, Texas, which is part of a large U.S. healthcare system. We will obtain preliminary data on the intervention’s acceptability, feasibility, and potential efficacy at the end of the study period. Additional insights will be gathered through interviews with key informants, who will be recruited from the hospital that is a main recruitment site, another hospital within the same healthcare system, and the surrounding community. Inclusion and exclusion criteria for eligible family caregivers and persons with dementia can be found in Table 2.

### Participants

#### Hospitalized persons with dementia

Inclusion criteria

- Aged 50 or older
- Has confirmed or suspected dementia or cognitive impairment
- Scores 4–6 on Functional Assessment Staging Tool [32]
- Admitted to the hospital from home
- English- or Spanish-speaking

#### Family Caregivers

Inclusion criteria

- Aged 18 or older
- English- or Spanish-speaking
- Identifies as the patient’s relative or partner who assists with medical care on a regular, unpaid basis

#### Key Informants: (Hospital Staff)

Inclusion criteria

- Aged 18 or older
- English-speaking
- Affiliated or employed full-time or part-time at two hospitals in Austin, Texas
- Involved directly or indirectly with patient care (i.e., nurses, physicians, physician assistants, care managers, social workers, chaplains, discharge planners, information technology staff, administrative managers).

#### Key Informants: Family Caregivers

Inclusion criteria

- Aged 18 or older
- English- or Spanish-speaking
- Currently or previously cared for a relative with dementia

#### Key Informants: Content Expects or Community Service Providers

Inclusion criteria

- Aged 18 or older
- English- or Spanish-speaking
- Identified as having expertise in dementia care or related areas (e.g., aging service providers, staff at Alzheimer’s Association, scholars in aging, dementia, caregiving, or care transition)

### Conceptual Model

Our CEDART intervention model was designed with an understanding of the dynamic, longitudinal, and often complex nature of care transitions. While the standardization of transitional care interventions may be useful in terms of replicability and scalability, effective interventions must be adaptable and individualized to each patient-caregiver dyad. Successful care transition interventions also anticipate and address emergent care needs, rather than strictly adhering to a predetermined care plan. Thus, our current model integrates key concepts from the Sociocultural Stress and Coping Model and Roberts’ Crisis Intervention Model to maximize effective intervention delivery and adaptability. The Sociocultural Stress and Coping Model [20] provides a comprehensive lens to examine the role of sociocultural influences and social determinants of health on persons with dementia and caregiver outcomes. Research on dementia caregiving, including our own work, suggests that factors such as caregiving network size, distrust of healthcare systems, and social determinants of health shape how persons with dementia and caregivers adjust to care transition processes and implement healthcare provider recommendations [33–38]. These factors can therefore significantly impact patient and caregiver outcomes. Accordingly, the community health worker interventionist must systematically assess contextual characteristics of patient-caregiver dyads to identify their unique support needs, potential barriers to accessing informal and formal services, and strategies to address any unmet needs.

Roberts’ Crisis Intervention Model [30] informs the overall process and steps of establishing rapport, as well as conducting assessments, care planning, and follow-ups with the family caregiver. Therefore, an effective intervention approach requires addressing each step in this framework, though caregivers may not follow these steps sequentially. For instance, a caregiver may revisit earlier stages of the model, such as exploring feelings, during later stages like planning. This flexibility is normal and does not reflect ineffective support. Given that continuous assessment throughout the intervention is critical, the community health worker is expected to regularly confirm whether caregivers’ evolving needs, priorities, and emotions are being addressed.

### Conceptual Model

Our CEDART intervention model was designed with an understanding of the dynamic, longitudinal, and often complex nature of care transitions. While the standardization of transitional care interventions may be useful in terms of replicability and scalability, effective interventions must be adaptable and individualized to each patient-caregiver dyad. Successful care transition interventions also anticipate and address emergent care needs, rather than strictly adhering to a predetermined care plan. Thus, our current model integrates key concepts from the Sociocultural Stress and Coping Model and Roberts’ Crisis Intervention Model to maximize effective intervention delivery and adaptability. The Sociocultural Stress and Coping Model [20] provides a comprehensive lens to examine the role of sociocultural influences and social determinants of health on persons with dementia and caregiver outcomes. Research on dementia caregiving, including our own work, suggests that factors such as caregiving network size, distrust of healthcare systems, and social determinants of health shape how persons with dementia and caregivers adjust to care transition processes and implement healthcare provider recommendations [33–38]. These factors can therefore significantly impact patient and caregiver outcomes. Accordingly, the community health worker interventionist must systematically assess contextual characteristics of patient-caregiver dyads to identify their unique support needs, potential barriers to accessing informal and formal services, and strategies to address any unmet needs.

Roberts’ Crisis Intervention Model [30] informs the overall process and steps of establishing rapport, as well as conducting assessments, care planning, and follow-ups with the family caregiver. Therefore, an effective intervention approach requires addressing each step in this framework, though caregivers may not follow these steps sequentially. For instance, a caregiver may revisit earlier stages of the model, such as exploring feelings, during later stages like planning. This flexibility is normal and does not reflect ineffective support. Given that continuous assessment throughout the intervention is critical, the community health worker is expected to regularly confirm whether caregivers’ evolving needs, priorities, and emotions are being addressed.

### Intervention

The CEDART intervention begins at hospital admission and continues as families transition home. The intervention can be adapted over time to reflect different transition pathways among persons with dementia (e.g., hospital to home vs. hospital to nursing home to home). The types and combinations of transitional support provided (e.g., education on dementia trajectory, information on applying for social services, access to transportation service) by the community health worker are informed by prior studies, including a 2024 systematic review of 126 transitional care interventions [16]. One medical school-affiliated hospital located in Austin, Texas will be the main study site for intervention delivery and dyad enrollment.

**Figure 2:**
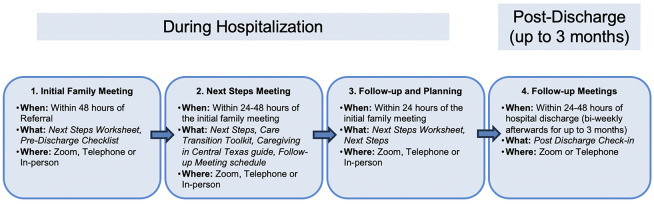
CEDART Intervention Process

The intervention is structured to address each hospitalized dyad’s needs through an initial assessment meeting, regular bi-weekly or weekly follow-ups, and facilitated connections to community resources. The complete intervention is designed to be delivered over three months following discharge. Figure 2 lists all intervention-related tools such as assessment and care planning forms used during each major step of the intervention process.

The intervention begins with an initial assessment conducted by the community health worker to identify primary and secondary caregiving stressors, currently utilized resources, social support, and additional needs related to social determinants of health, dementia education, care navigation, and caregiver health. This initial assessment meeting, lasting approximately 45–60 minutes, will occur either in person, via Zoom, or by telephone. It may take place during the hospital stay or within one week after discharge. The community health worker will use the initial assessment to inform the development of an individualized post-discharge care plan. They will also contact the family caregiver to review and, if needed, revise the plan to address any emerging needs or concerns. If the caregiver identifies urgent healthcare questions or requests assistance in communicating with hospital staff about patient care or discharge planning, the community health worker will facilitate communication as promptly as possible. Dyads will also receive a dementia caregiving toolkit during this meeting, which includes an individualized list of community resources.

Within the first week after discharge, the community health worker will meet with the dyad via phone or Zoom to review caregiving goals, assess additional support needs—such as care navigation and access to social services—and address any emerging concerns.

During the first month post-discharge, the community health worker will meet with the caregiver via Zoom or telephone on a weekly or bi-weekly basis to provide coaching, dementia caregiving education, and care navigation support. In the second and third months, they will conduct approximately four additional bi-weekly follow-up meetings with the caregiver. These sessions, lasting 30–45 minutes, will be conducted via telephone or Zoom. Follow-up meetings will include a brief review of the dyad’s goals and address any new questions or needs related to post-discharge care navigation. The community health worker will also continue to monitor the dyad’s well-being, offer socioemotional support, and facilitate connections to community resources as needed.

### Control group

This study will employ a single-arm feasibility design without a control group.

### Feasibility study outcomes

Primary study outcomes will be focused on feasibility, acceptability, and fidelity. Secondary study outcomes include assessing the acceptability of measurement tools administered during caregiver interviews at baseline, 6-weeks, and 12-weeks, and exploring the preliminary efficacy of the intervention.

Main outcomes for our study focus on feasibility and include:

1. Number of participants with dementia and caregivers screened, eligible, and enrolled.
2. Identification of effective recruitment strategy to inform a future definitive trial.
3. Feasibility and acceptability of the intervention, including its content, delivery, and fidelity.
4. Completion rates of follow-up interviews and outcome measures.
5. Reasons for non-recruitment and attrition.
6. Acceptability of recruitment procedures, assessments, data collection tools, and intervention delivery.
7. Baseline scores and variability of secondary outcomes to inform sample size estimates for a future trial.

### Qualitative data collection

#### Design

In addition to quantitative metrics, we will also use a qualitative descriptive approach [39, 40] to describe stakeholders’ views on the acceptability and feasibility of the CEDART intervention procedures, recruitment process, content and delivery, and data collection methods. This approach will help identify factors that facilitated or hindered study participation. Any additional feasibility issues that may emerge from data analysis will inform the design of a future randomized controlled trial. Table 3 summarizes who will be interviewed throughout the study period.

Qualitative data collection will consist of individual, semi-structured key informant interviews with diverse stakeholder groups from two hospitals, and the broader community. The interviews will occur both before and after intervention implementation to capture stakeholder insights regarding the transitional care process, identify existing challenges, and elucidate priorities for enhancing care transitions. All qualitative interviews will last approximately 30–60 minutes and will be conducted via each participant’s preferred method, including in-person, telephone, or Zoom.

### Main trial outcome measurements

To inform the design of a future definitive trial, we will evaluate the feasibility and acceptability of several secondary outcome measures. These include the Quality of Life in Alzheimer’s Disease scale [41, 42] and caregiver-reported 30- and 90-day hospital and emergency department (ED) readmissions to assess impact on persons with dementia. Caregiver-specific outcomes will be measured using the Zarit Burden Interview–4 [43], Caregiver Outcomes of Psychotherapy Evaluation [44], Family Caregiver Activation in Transitions (FCAT) [45], and indicators of post-discharge care compliance [46]. Detailed descriptions of these measures are attached [see Additional File 3]. Table 4 summarizes how feasibility study outcomes will inform a future definitive trial.

### Adverse events

For this study, an adverse event (AE) is any unexpected occurrence that risks participants’ safety, well-being, or confidentiality. Given the study’s focus on interviews, surveys, and a psychosocial intervention for caregivers of persons with dementia, AEs may include:

1. Psychological or Emotional Distress: Significant distress triggered by study participation.
2. Breach of Confidentiality: Unintended disclosure of participant information.
3. Participant Complaints or Concerns: Reports of coercion, dissatisfaction, or procedural issues.
4. Intervention-Related Issues: Increased stress, burden, or unintended negative consequences from interacting with the community health worker.
5. Safety Concerns: Signs of self-harm, harm to others, or elder abuse requiring mandated reporting.

All AEs will be documented and assessed by the study team. Participants experiencing distress will be offered support, with the option to pause or withdraw. If further intervention is needed (e.g., suspected abuse or severe psychological distress), appropriate reporting and referral protocols will be followed.

### Sample size

As this is a single-arm pilot study, a power analysis was not required. It is suggested that sample sizes for pilot studies be determined based on practical factors such as participant availability, budget constraints, and the number of participants required to sufficiently assess feasibility outcomes [47]. Based on these considerations, a target sample size of 40 caregiver-patient dyads was selected. This sample size accounts for the estimated number of weekly admissions of patients with dementia at the recruiting hospital and the anticipated recruitment timeline.

### Recruitment

Research team members and the main recruitment hospital site providers (e.g., hospitalists, resident physicians, case managers) will conduct initial screenings of potentially eligible patients by reviewing electronic medical record multiple times per week. Given that the Functional Assessment Staging Tool scale is not routinely documented at the hospital, patients with medical record findings clearly indicating a Functional Assessment Staging Tool score above six (e.g., loss of verbal response, independent walking, or smiling not due to another medical condition) will be deemed ineligible [32]. If no such findings are documented, research assistants will administer the Functional Assessment Staging Tool scale to caregivers for eligibility determination.

For enrollment, hospital clinical providers (e.g., primary care, palliative care, and case management teams) may introduce the study to eligible caregivers or caregiver-patient dyads. If interested, the provider will notify the research team, who will then contact the caregiver to schedule an in-person or virtual meeting to review study details, confirm eligibility, and obtain consent. If a clinical provider does not introduce the study to the caregiver, the research team may reach out via phone call or in-person visit to introduce the study following confirmation from the case management team that the family may be a good fit. The research team will:

- Confirm eligibility.
- Obtain caregiver consent.
- Seek caregiver permission to contact the patient for consent/assent.
- Assess the patient’s capacity to consent/assent and obtain consent or proxy consent as needed.

Recruitment of clinical providers and hospital staff for interviews will occur via word-of-mouth, email, and snowball sampling. The research team will collaborate with administrative and clinical leadership to share study information with relevant staff (e.g., case managers, social workers, palliative care, and primary medical team) through emails, internal hospital communications, and team meetings. Study details will also be disseminated via a study flyer.

Stakeholder recruitment for key informant interviews will follow a similar approach, leveraging professional networks, service provider referrals, and internet searches. Caregivers for persons with dementia screened for participation in the intervention who do not meet eligibility criteria may also be considered for recruitment as key stakeholders should they express desire to contribute to the study in another way. Potential participants will be contacted via email or phone, and those expressing interest will receive further study details before providing consent. Snowball sampling will be used to expand recruitment by encouraging participants to refer colleagues. To promote participant retention, all participating family caregivers will receive monetary compensation (up to $90USD) based on intervention completeness.

### Criterion for progression to a definitive randomized controlled trial

The decision to proceed with a future definitive randomized controlled trial will depend on meeting the established criteria for each feasibility outcome as shown in Table 5.

### Data management

All participant data will be kept strictly confidential. Qualitative interview data will be digitally recorded, transcribed, and de-identified after verification for accuracy. Quantitative data (e.g., survey responses, chart reviews) will be entered into an SPSS dataset without identifiable information. Each participant will be assigned a unique study ID, with identifiable information (e.g., name, contact details) stored separately from research data. Identifiable information will be maintained in a password-protected Microsoft Excel file on a secure server until study completion, accessible only to authorized research personnel. Protected health information from chart reviews will be stored separately from participant identifiers after data entry into a secure, password-protected database. Both electronic and physical copies of protected health information will use study IDs. Hard copies of data will be kept in locked file cabinets within a secured office. Electronic data will be stored in a password-protected database. Audio recordings will be erased after transcription.

### Data analysis

#### Quantitative analysis

All quantitative data analyses (e.g., descriptive statistics, correlations, and linear mixed-effects models) will be conducted using SPSS v24 and RStudio software. To assess the intervention’s potential impact on dyad outcomes, we will perform within-group, longitudinal analyses following an intention-to-treat approach. Changes in outcomes from baseline to 6-week and 12-week follow-up will be examined using linear mixed-effects models to account for repeated measures. A change of 0.3 standard deviation units or greater in key outcome measures will be considered indicative of potential efficacy, consistent with effect sizes observed in similar palliative care pilot studies. Because the nature of the study is exploratory, analysis will focus on in-sample effect size estimation rather than formal hypothesis testing. Statistical power is not a primary concern as no inferential statements will be made, which is consistent with appropriate analytical approaches for pilot study data. To analyze the acceptability and feasibility of the protocol, as well as barriers to recruitment and retention, we will use analytic techniques for mixed methods study such as content analyses and descriptive methods.

#### Qualitative analysis

All qualitative interviews will be digitally recorded, transcribed verbatim by a professional transcription service, and uploaded into Dedoose (online software for qualitative data management) for analysis. A coding scheme and codebook will be developed, incorporating both externally generated codes based on the conceptual framework and internally generated codes emerging from the analysis. All codes will focus on identifying the challenges, acceptability and feasibility of the intervention, as well as opinions on session length, frequency, and delivery methods. Member checking will be conducted throughout the interview process. To enhance trustworthiness, an audit trail will be maintained, and the research team will regularly review and provide critical feedback on emerging codes. Analysis will involve examining patterns within and across coded texts to identify converging themes and reach consensus on key findings.

### Fidelity

Intervention adherence and fidelity will be assessed on an ongoing basis. Between 25–50% of the audio recordings of initial assessments and follow-up assessment meetings will be reviewed using the checklist [see Additional file 4]. This checklist provides a rating of the quality of the community health worker’s intervention delivery with regards to their core competencies, actions, and skills. The assessment and follow-up forms that correspond with the audio recordings will be reviewed for accuracy and completeness. The review will be guided by the intervention fidelity checkpoint lists and guides for addressing potential problems and offering appropriate solutions. A score of 35 and higher of the fidelity checklist assessment will be considered to meet fidelity requirements and adherence. All checklist scores and findings will be shared with the community health worker to improve intervention delivery and identify areas for improvement.

### Modifications

Initially, the community health worker was required to complete the initial assessment with the family caregiver prior to their discharge. However, due to the dynamic, unpredictable nature of hospital discharge processing and short length of stay in the hospital among many patients, the protocol was changed to allow the community health worker to complete the initial assessment meeting during hospitalization or within the first week of discharge.

### Research Advisory Committee

We will establish a Research Advisory Committee composed of professionals, service providers, and caregivers to provide expert feedback on stakeholder priorities and meaningful outcomes in care transitions. The committee will offer recommendations on intervention content, delivery methods, community health worker training, fidelity standards, and research protocols. The committee will help ensure that the needs and priorities of diverse stakeholders, including persons with dementia, caregivers, and healthcare systems, are addressed. Additionally, they will support future planning by identifying funding mechanisms for the future definitive randomized controlled trial and contributing to the development of the study approach.

### Ethics

Ethical approval was obtained from the Institutional Review Board (IRB) at the University of Texas at Austin (IRB ID: STUDY00004286-MOD05). All intervention modifications will be submitted and approved by IRB prior to implementation.

## Discussion

Despite evidence that persons with dementia face increased risks of morbidity and mortality after hospital discharge [51], few interventions have been designed to support hospital-based care transitions for this population [9]. Existing interventions often rely on complex protocols involving multiple modes of support [13, 52]. CEDART addresses this gap by utilizing community health workers to deliver a straightforward, replicable intervention for family caregivers of persons with dementia during hospital-to-community transition. This study will assess its feasibility, acceptability, and preliminary efficacy. If feasibility is demonstrated, findings will inform the development of a future randomized controlled trial.

### Study status

Recruitment began in January of 2024 and will end in May of 2025.

## Figures and Tables

**Table 1.** Schedule of enrollment, intervention and assessments per SPIRIT 2013

	Study Period		
	Enrollment	Post Enrollment	Close Out (12 weeks)
Timepoint	-T1	T1	T2
Enrollment			
Eligibility screen	X		
Informed consent	X		
Demographics	X		
Intervention			
Community health worker-led Care Transition Support		X	
Assessments			
QoL-AD, number of hospital and emergency department admission, ZBI-4, COPE, FCAT, Post-discharge compliance	X	X	X
Acceptability interview			X

Note: QoL-AD = Quality of Life in Alzheimer's Disease; ZBI-4 = Zarit Burden Interview–4; COPE = Caregiver Outcomes of Psychotherapy Evaluation; FCAT = Family Caregiver Activation in Transitions. Post-discharge compliance refers to caregiver-reported adherence to the recommended post-hospital care plan.

**Table 2.** Patient and caregiver eligibility criteria

Patient	Caregiver

Inclusion Criteria	Inclusion Criteria
1. ≥50 years of age 2. Confirmed or suspected dementia or cognitive impairment 3. Scores 4–6 on Functional Assessment Staging Tool 4. Admitted to the hospital from home 5. English- or Spanish-speaking	1. ≥18 years of age 2. English- or Spanish-speaking 3. Patient’s relative or partner 4. Provides regular, unpaid medical care and or assistance
Exclusion Criteria	Exclusion Criteria
1. Do not meet all inclusion criteria 2. Patients who are eligible for hospice	1. Do not meet all inclusion criteria

**Table 3.** Qualitative data collection process

Participants	Number interviewed before the start of the study	Number interviewed at post-discharge Week 12	Number interviewed at the end of the study
Caregiver intervention participants		40	
Community health worker interventionist			1
Hospital providers	5		5
Caregivers	5		5
Community service providers and content experts	5		5

**Table 4.** Summary overview of how the feasibility study may inform a future definitive trial

Pilot study	Future definitive trial
Intervention content and delivery	Finalized Intervention content and modes of delivery
Participant recruitment	Effective participant recruitment strategy established
Training requirements	Research staff and CHW training needs identified
Study protocol	Study protocol developed for all study processes
Data collection tools (quantitative & qualitative)	Final quantitative secondary outcome measurements selected, and qualitative interview guides developed
Fidelity assessment procedures	Fidelity assessment measures and procedures finalized
RCT protocol	RCT protocol developed

**Table 5.** Feasibility outcomes and criteria for advancing to a future definitive randomized trial

Feasibility outcomes	Criteria for determining success of feasibility to progress to a future definitive randomized trial
Number of people with dementia and caregivers screened, deemed eligible, and enrolled in the study.	Recruitment of at least 25% of eligible participants (people with dementia and caregivers).
Identification of the most effective participant recruitment strategy for a future definitive trial.	Identification of the most effective recruitment strategy, informed by recruitment rates and qualitative insights from key stakeholders.
Feasibility and acceptability of the intervention, including content, delivery, and fidelity assessments.	Over 70% of participants attend at least 70% of intervention sessions, with the intervention delivered according to feasibility and fidelity targets (ensuring adherence to content, frequency, and quality guidelines outlined in the intervention manual).
Follow-up rates and completion of outcome measures	Less than 30% participant attrition at follow-up.
Reasons for non-recruitment, or participant attrition.	Compilation of reasons for non-recruitment, non-adherence, or attrition based on qualitative data from participants.
Acceptability of recruitment procedures, assessments, data collection tools, and intervention delivery.	Recruitment processes, assessments, data collection tools, and intervention delivery deemed acceptable by more than 70% of stakeholders and study participants.
Baseline scores and variability of secondary outcome measures to inform sample size estimates for a future trial.	Sample size estimates for persons with dementia and caregivers determined using baseline scores of primary outcomes: person with dementia quality of life (QoL-AD), caregiver caregiving efficacy (COPE), and caregiver burden (ZBI-4). Variability in secondary outcomes remains within acceptable limits for sample size calculations and potential attrition considerations.

Note: QoL-AD = Quality of Life in Alzheimer’s Disease; COPE = Caregiver Outcomes of Psychotherapy Evaluation; ZBI-4 = Zarit Burden Interview–4.

## Data Availability

The datasets generated and analyzed during the study will be available from the corresponding author upon reasonable request.

## Abbreviations

AE: Adverse Events
CEDART: Connecting and Empowering Persons Living with Dementia and Caregivers During Transition
SPSS: Statistical Package for Social Sciences
